# *In silico* analysis of bacterial arsenic islands reveals remarkable synteny and functional relatedness between arsenate and phosphate

**DOI:** 10.3389/fmicb.2013.00347

**Published:** 2013-11-20

**Authors:** Hang Li, Mingshun Li, Yinyan Huang, Christopher Rensing, Gejiao Wang

**Affiliations:** ^1^State Key Laboratory of Agricultural Microbiology, College of Life Sciences and Technology, Huazhong Agricultural UniversityWuhan, P. R. of China; ^2^Department of Plant and Environmental Sciences, University of CopenhagenFrederiksberg, Denmark

**Keywords:** arsenic islands, arsenite oxidase, AioBA, phosphorus, synteny

## Abstract

In order to construct a more universal model for understanding the genetic requirements for bacterial AsIII oxidation, an *in silico* examination of the available sequences in the GenBank was assessed and revealed 21 conserved 5–71 kb arsenic islands within phylogenetically diverse bacterial genomes. The arsenic islands included the AsIII oxidase structural genes *aioBA*, *ars* operons (e.g., *arsRCB*) which code for arsenic resistance, and *pho*, *pst*, and *phn* genes known to be part of the classical phosphate stress response and that encode functions associated with regulating and acquiring organic and inorganic phosphorus. The regulatory genes *aioXSR* were also an island component, but only in *Proteobacteria* and orientated differently depending on whether they were in α-*Proteobacteria* or β-/γ-*Proteobacteria*. Curiously though, while these regulatory genes have been shown to be essential to AsIII oxidation in the *Proteobacteria*, they are absent in most other organisms examined, inferring different regulatory mechanism(s) yet to be discovered. Phylogenetic analysis of the *aio*, *ars*, *pst*, and *phn* genes revealed evidence of both vertical inheritance and horizontal gene transfer (HGT). It is therefore likely the arsenic islands did not evolve as a whole unit but formed independently by acquisition of functionally related genes and operons in respective strains. Considering gene synteny and structural analogies between arsenate and phosphate, we presumed that these genes function together in helping these microbes to be able to use even low concentrations of phosphorus needed for vital functions under high concentrations of arsenic, and defined these sequences as the arsenic islands.

## Introduction

Microbial arsenite (AsIII) oxidation converts the more toxic AsIII to the less toxic AsV which is known to be catalyzed by a molybdenum-containing enzyme. The AsIII oxidase enzyme is encoded by *aioBA* (previously referred to as *aoxAB*) (Silver and Phung, 2005). Putative AioA were found to be specific for AsIII-oxidizing bacteria, therefore the usefulness of the *aioBA* as a functional marker indicating the ability to oxidize AsIII of a strain was proposed (Inskeep et al., 2007; Quéméneur et al., 2008; Hamamura et al., 2009). However, *aioA* is not a suitable marker for microbial diversity studies because its phylogeny does not always strictly correlate with that of the 16S rRNA genes, due to horizontal gene transfer (HGT) (Heinrich-Salmeron et al., 2011). In addition, in some AsIII-oxidizing strains, an *aioA* sequence was not detected either by PCR nor genome sequencing (Richey et al., 2009). It is now known that a new type of AsIII oxidase gene *arxA*, which was only distantly related to *aioA*, could be identified in a number of strains (Zargar et al., 2010, 2012).

Currently, the genetics underlying AsIII oxidation and its regulation are perhaps best understood in *Agrobacterium tumefaciens* 5A. The *aioBA* genes are part of the *aioX-aioS-aioR-aioB-aioA-cytc2-chlE* operon that has been shown to be regulated by a two-component regulatory pair comprised of the sensor kinase AioS and its cognate response regulator AioR in conjunction with the periplasmic AsIII-binding protein AioX (Kashyap et al., 2006; Koechler et al., 2010; Liu et al., 2012). In addition, the σ^54^ factor (RpoN) has been shown by two different groups to play a role in *aioBA* regulation (Koechler et al., 2010; Kang et al., 2012). A −24/−12 box for RpoN binding has been detected upstream of *aioB* and shown to be important for *aioBA* expression by 5′ RACE (rapid-amplification of cDNA ends) (Sardiwal et al., 2010) and precision deletion experiments (Koechler et al., 2010). Furthermore, AioR also contains a conserved domain for response regulators that could regulate σ^54^-type promoters (Sardiwal et al., 2010). RpoN is viewed to form a close complex with RNA polymerase, which requires energy provided by regulators for transcriptional initiation. The σ^54^-dependent regulators such as AioR may bind to upstream activation sequences (UAS) of σ^54^-type promoters for energy conservation (Shingler, 2011).

Regarding sequences in the vicinity of the *aio* operon, Silver and Phung (2005) first proposed the concept of an “arsenic island” based on a 71-kb DNA region of the *Alcaligenes faecalis* genome which contains over 20 functionally related genes such as those encoding AsIII oxidase AioBA, ArsAB for AsIII efflux, and a variety of oxyanion ABC transporters. Muller et al. (2007) reported the gene sequences in the vicinity of *aioBA* in *Herminiimonas arsenicoxydans* ULPAs1 and several other strains. Later, Arsène-Ploetze et al. (2010) proposed that *aioBA* was located in a genomic island which may have been acquired by HGT in *Thiomonas* sp. 3As. However, since only a few *aio* operons were known until recently, the definition and distribution of such arsenic islands was unclear and speculative. Due to the development and usage of high-throughput sequencing, more *aio* operons could be identified in microbial genomes (Hao et al., 2012; Huang et al., 2012; Li et al., 2012; Lin et al., 2012; Phung et al., 2012). From visual inspection, gene patterns associated with the *aioBA* genes became apparent and warranted a more detailed characterization.

One such pattern is the frequent physical association of genes involved with As and phosphorus (P) metabolism (Moreno-Sanchez et al., 2012). As and P are both members of Group 15 on the periodic table, resulting in their being structural analogs, such that AsV and phosphate may be co-metabolized, with the best examples involving AsV substituting for phosphate as substrate for phosphate transporters or interfering with ATP metabolism (Moreno-Sanchez et al., 2012). The Pho regulation is induced by P starvation and has been reported to control about 30 genes in 9 transcripts, including *phnCDEFGHIJKLMNOP* genes for phosphonate assimilation, *phoE* for outer membrane phosphoporin, *phoA* for alkaline phosphatase, *pstSCAB* genes for specific phosphate transport and *ugpABCD* genes for glycerol-3-phosphate transporter (Hsieh and Wanner, 2010). Recently, Kang et al. (2012) demonstrated that in *A. tumefaciens* strain 5A the close genomic association of the *aioXSRBA* genes with genes coding for functions involved with acquiring P under P-stress conditions is not simply coincidental. Surprisingly, induction of *aioBA* is repressed under high phosphate conditions and involves regulatory components of the phosphate stress response. Either or both PhoB response regulators, PhoB1 and PhoB2, are required for normal transcriptional kinetics of *aioBA* and *aioSR*. In addition, genes usually regulated by environmental phosphate levels, *pstS1* and *phoU*, were found to be regulated by ArsR in an AsIII-dependent manner (Kang et al., 2012).

The primary aim of this study was to characterize the physical arrangement and functional relatedness of *aio*, *pho*, *pst*, and *ars* genes among the available arsenic islands. In addition, we also examined the phylogenetic relationships of these genes so as to assess mode of inheritance and used this information to begin assimilating a broader picture of how AsIII oxidation is regulated in different organisms and speculate the functional relationships of some genes that appear to have been repeatedly co-inherited in nature.

## Results

### Detection of genes in the vicinity of *aio* operon revealed putative arsenic islands

**Figure 1:**
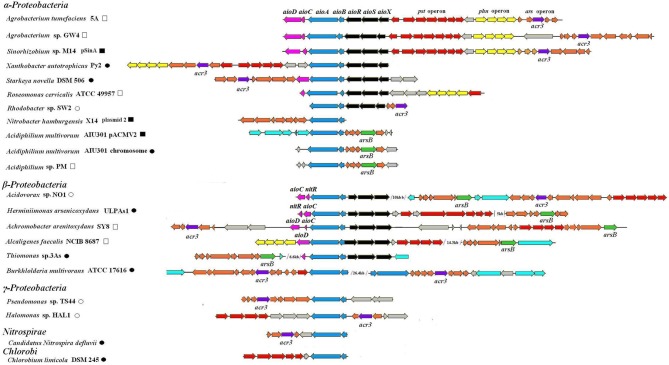
**Gene arrangements of the 21 arsenic islands.** Arrows with different colors represent the following genes: blue for *aioBA*, black for *aioXSR*, pink for *aioCD or nitR (encoding* cytochrome c and molybdenum biosynthesis protein or nitroreductase, respectively), red for *pst* operon, yellow for *phn* operon, orange for *ars* operon in which green for *arsB*, purple for *acr3*, light blue for mobile element. ■ and □ represent the reported and predicted plasmid-originated sequences, respectively. • and ° represent the reported and predicted chromosome-originated sequences, respectively. GenBank accession numbers are as follows: *Acidovorax* sp. NO1 (AGTS01000000), *Herminiimonas arsenicoxydans* ULPAs1 (CU207211), *Alcaligenes faecalis* NCIB 8687 (AY297781), *Achromobacter arsenitoxydans* SY8 (AGUF01000000), *Sinorhizobium* sp. M14 (GU990088), *Agrobacterium* sp. GW4 (JQ423942), *Roseomonas cervicalis* ATCC 49957 (NZ_ADVL01000677), *Xanthobacter autotrophicus* Py2 (NC_009720), *Rhodobacter* sp. SW2 (NZ_ACYY01000001), *Starkeya novella* DSM 506 (NC_014217), *Nitrobacter hamburgensis* X14 (NC_007960), *Halomonas* sp. HAL1 (EU651834), *Pseudomonas* sp. TS44 (EU311944), *Candidatus nitrospira defiuvii* (NC_014355), *Chlorobium limicola* DSM 245 (CP001097), *Burkholderia multivorans* ATCC17616 (NC_010087), *Acidiphilium multivorum* AIU301 (NC_015186 and NC_015187).

A total of 57 full-length *aioBA* operons encoding arsenite oxidase were detected in 55 strains using a BLAST search in GenBank. Among them, genes in the vicinity of the *aioBA* operons showed significant synteny among 21 genome sequences (ranging 5–71 kb). These genes that were all responsible for AsIII oxidation (21 *aio* operons, *aioBA*, or *aioXSRBACD*), arsenic resistance (23 *ars* operons, e.g., *arsR, arsC*, *arsB*, and *acr3*), phosphate transport (10 *pst*1 operons, e.g., *pstS, pstC, pstA*, and *pstB*) and phosphonate transport (6 *phn*1 operons, e.g., *phnC, phnD, phnE*, and *phnE*) were frequently detected (Figure 1). Considering the major function for AsIII's oxidation, we refer to these 21 sequences as arsenic oxidase gene islands (Figure 1). In some closely related bacterial strains, gene arrangements of the islands showed excellent synteny as similar gene and operon arrangements were found in *A. tumefaciens* 5A, *Agrobacterium* sp. GW4 and *Sinorhizobium* sp. M14. In addition, the same arrangement was also shared by *Acidiphilium multivorum* AIU301 and *Acidiphilium* sp. PM. However, other distantly related bacteria containing an arsenic island did not show a similar arrangement (Figure 1). Later on, *Agrobacterium albertimagni* strain AOL15 (Trimble et al., 2012a) and *Achromobacter pichaudii* strain HLE (Trimble et al., 2012b), were sequenced, but showed similar arsenic gene islands to *A. arsenitoxydans* SY8 (Li et al., 2012) and *A. tumefaciens* 5A, respectively (Hao et al., 2012) (data not shown).

By scanning the genomes, we found that *aio* operons were only present as a single copy and often located within the arsenic islands. To better interpret the results of this analysis, it is important to point out that in addition to the *pst* or *phn* operons on the arsenic islands (here referred to as *pst*1 and *phn*1), almost all strains possessed another *pst* or *phn* operon located distantly (here referred to as *pst*2 or *phn*2). The phylogenies of the representative amino acid sequences (AioA, PstS, and PhnC) for *aio*, *pst* and *phn* operons were compared to their 16S rDNAs in order to determine whether possible HGT had taken place. Since *ars* operons have frequently been shown to associate with HGT events (Tuffin et al., 2005; Cai et al., 2009a), only the representative *ars* genes (*acr3* or *arsB*) on the arsenic islands were analyzed in this study (see following sections for detailed results), although there are orthologs in many other phyla.

### The arsenic islands are localized on chromosomes or on plasmids

Of the 21 arsenic islands analyzed in this study, 3 have been shown to be localized on a plasmid (GenBank GU990088, CP000321 and AP012037) and 8 on a chromosome (GenBank CP000781, CP003126, AP012037, NC_009138, FP475956, NC_010087, FP929003, and CP001097) (Figure 1). To determine where all of the 21 arsenic islands are located, we performed a bioinformatics analysis to predict localization on a plasmid using the cBar program (Zhou and Xu, 2010). According to our analysis, nine arsenic islands were predicted to be located on a plasmid and 12 on a chromosome (Figure 1). The previously determined localization of 3 arsenic islands on a plasmid and eight on a chromosome were all correctly predicted, demonstrating good reliability of plasmid prediction using cBar. The plasmid-borne arsenic islands were prevalent in *α-Proteobacteria* (7/11). Notably, strains *Agrobacterium* sp. GW4, *A. tumefaciens* 5A, and *Sinorhizobium* sp. M14 shared similar “arsenic island” arrangements, all predicted to be located on a plasmid. Again, these predictions were in agreement with the known localization of the arsenic gene island on plasmid pSinA (GenBank GU990088) of strain *Sinorhizobium* sp. M14 (Figure 1). Furthermore, the three arsenic islands from *Acidiphilium multivorum* AIU301 and *Acidiphilium* sp. PM had a similar arrangement of their genes and were all localized on a plasmid (Figure 1), two of the arsenic islands were predicted by cBar whereas one occurs on pACMV2 (AP012037). It appears likely that these strains may have acquired their respective arsenic islands by HGT.

### Wide spread distribution and genomic stability of aioBA

**Figure 2:**
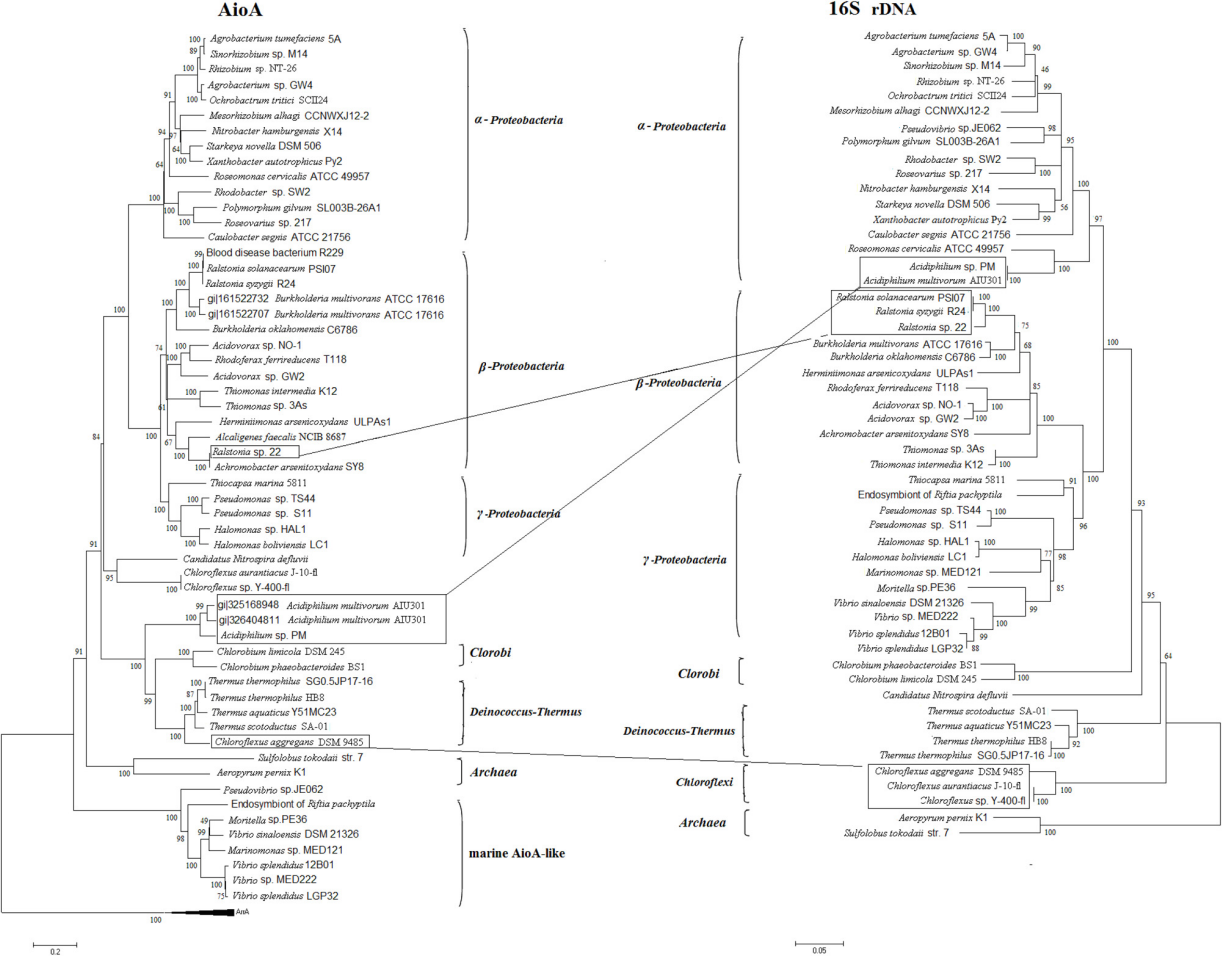
**The neighbor-joining (NJ) phylogenetic trees of AioA sequences and 16S rDNA sequences.** Putative horizontal gene transfer events (labeled with frames) have been compared based on the inconsistency of AioA amino acid tree (on the left) and the 16S rDNA tree (on the right).

The phylogenetic tree of AioA was generally in accordance with the 16S rDNA phylogeny which branched into *Proteobacteria, Chlorobi, Deinococcus-Thermus, Chloroflexi*, and *Archaea* (Figure 2), and the AioBA phylogeny was similar to that of AioA (data not shown). Most of the sequences encoding AioA were found in *Proteobacteria*, and mainly be divided into two groups. Group I is made up of sequences of α-*Proteobacteria* and Group II is comprised of β- and γ-*Proteobacteria*. This distribution was consistent with a previous analysis in which partial AioA sequences obtained by degenerate primers was distributed along a similar pattern (Heinrich-Salmeron et al., 2011). However, eight AioA-like proteins from marine α- or γ-*Proteobacteria* clustered together into a separate branch and exhibited a unique arrangement. The genes encoding this subfamily of AioA were all located downstream of two genes encoding the cytochrome c peroxidase MauG and were arranged in the gene order of *mauG-mauG-aioBA*. This phylogenetically distinct clade of these AioA-like proteins may have evolved in their marine environment due to unique conditions. Compared to the 16S rDNA phylogenetic tree, there were three conflicts with the AioA phylogenetic tree, which suggests the occurrence of HGT. For example, the two AioAs in *Acidiphilium multivorum* AIU301 (one located from the chromosome and one from a plasmid) and the AioA in *Acidiphilium* sp. PM fell into a clade together with *Chlorobi* and *Deinococcus-Thermus*, respectively (Figure 2). The *Chlorobi* or *Deinococcus-Thermus* strains appears to had transferred the *aioA* into *A. multivorum* AIU301 and *Acidiphilium* sp. PM since they all have been isolated from a similar acidic environment (San Martin-Uriz et al., 2011). In addition, a HGT might also be more likely since the two AioAs in *A. multivorum* AIU301 and *Acidiphilium* sp. PM were also predicted by cBar to be located on plasmids (Figure 1). *Chlorflexus aggregans* DSM 9485, *Chloroflexus* sp. J-10-fl and Y-400-fl are closely related based on 16S rDNA analysis, while the AioA of *C. aggregans* DSM 9485 clustered with the AioAs from *Deinococcus-Thermus* (Figure 2). It appears that *A. arsenitoxydans* SY8 had transferred *aioA* into *Ralstonia* sp. 22 by HGT (Lieutaud et al., 2010), and here again we found that the *aioA* of *A. arsenitoxydans* SY8 is located on plasmid.

### Analysis of the putative regulators for *aio*BA operons suggested different mechanisms of *aio* regulation

The genes encoding regulators AioXSR located upstream of *aioBA* were only identified in 12 strains of *Proteobacteria* among the 21 analyzed strains encoding *aioBA* as part of their arsenic island. All of these 12 strains belonged to either α-*Proteobacteria* or β-*Proteobacteria* (Figure 1). The transcriptional orientation of *aioXSR* genes differed between α- and β-*Proteobacteria*, which is in agreement with the AioA phylogeny (Figure 2). The *aioXSR* genes from α-*Proteobacteria* displayed the same transcriptional orientation as *aioBA*, while those from β-*Proteobacteria* displayed the opposite orientation (Figure 1). The other nine sequences without *aioXSR* genes were distributed in different taxonomic groups such as *Proteobacteria*, *Chlorobi* and *Nitrospriae*. In some of these identified species such as *Pseudomonas* sp. TS44 and *Halomonas* sp. HAL1, AsIII oxidation could be verified (Cai et al., 2009b; Lin et al., 2012). The mode and mechanism regulating expression of *aioBA* in these strains is unknown but might involve distantly located regulators. It is interesting that there are *nitRs* (encoding nitroreductases) after the *aioC* instead of *aioD* in *Acidovorax* sp. NO1 (AGTS01000000), *Herminiimonas arsenicoxydans* ULPAs1 (Figure 1). Recently, a disruption of the *nitR* in strain NO1 resulted in the delay of AsIIII oxidation indicating that the *nitR* may participate to the electron transfer in the strain (data not shown).

### Phylogenetical analyses of arsenite efflux proteins ArsB or ACR3 encoded in *ars* operons of the arsenic islands.

A total of 23 *ars* operons were detected in the arsenic islands and their inheritance models were analyzed by phylogenetic analyses of ArsB or ACR3, and comparing their phylogeny with those obtained using 16S rDNA. Among the 23 *ars* operons, eight ArsB and 13 ACR3 were detected. Some *ars* operons without the *arsB* or *acr3* genes such as those from *N. hamburgensis* X14 and *A. arsenitoxydans* SY8 could not be analyzed in this context.

Phylogenetic analysis suggested most ArsB arsenite efflux proteins were congruent with 16S rDNAs (Supplementary materials, Figure S1). However, a notable exception included the ArsBs from *Acidovorax* sp. NO1, *Thiomonas* sp. 3A and *A. faecalis* NCIB8687, which clustered together. All of these ArsB proteins were encoded as part of a transposon, again indicating HGT events by transposon insertion accounted for acquisition of these *ars* operons in the respective arsenic islands.

The ACR3s separated into two clades in previous studies (Achour et al., 2007) and we also found that ACR3s on the arsenic islands that could be divided into ACR3 (1) and ACR3 (2). In the respective ACR3 clades, their phylogenies were both in accordance with 16S rDNA phylogeny, therefore, suggesting genomic stability (Figure S2).

### Phylogenetic analyses of the phosphorus related *pst* and *phn* operons

**Figure 3:**
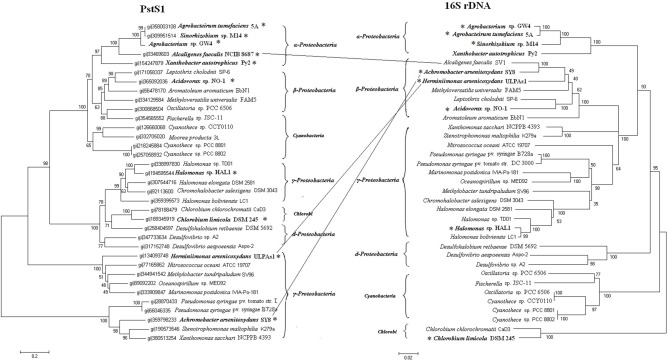
**Phylogenetic trees based on PstS1 sequences and 16S rDNA sequences.** Bold and ^*^symbol represent proteins from the strains of the arsenic islands while the others are not. Putative horizontal gene transfer events (connected lines) have been compared based on the inconsistency of the amino acid sequence tree (on the left) and the 16S rDNA tree (on the right).

The *pst*1 or *phn*1 genes are localized within arsenic islands, while *pst*2 and *phn*2 genes are localized distantly on the respective chromosomes. Phylogenetic analysis indicated that all of the Pst2 branched in accordance with the 16S rDNAs (Figure S3). Therefore, *pst*2 operons appear to follow vertical inheritance. However, Pst1 did not strictly branch as the phylogenetic tree based on the 16S rDNA sequences (Figure 3). The Pst1 of *Alcaligenes faecalis* NCIB 8687 (β-*Proteobacteria*) clustered together with the Pst1 of α-*Proteobacteria* strains *Agrobacterium tumefaciens* 5A, *Agrobacterium* sp. GW4, *Sinorhizobium* sp. M14 and *Xanthobacter autotrophicus* Py2. The Pst1 of *A. arsenitoxydans* SY8 and *H. arsenicoxydans* ULPAs1 (β-*Proteobacteria*) were more related to those from γ-*Proteobacteria*. These results suggest that HGT may have occurred in transmission of the *pst*1 operon.

**Figure 4:**
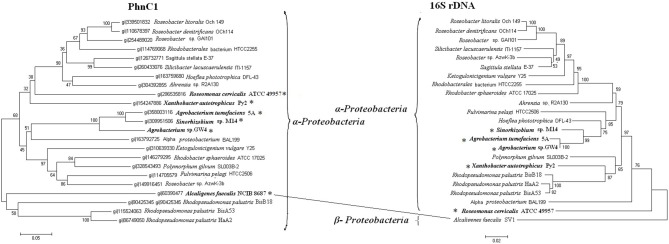
Phylogenetic trees of PhnC1 and 16S rDNA sequences. Bold and ^*^symbol represent proteins from the strains of the arsenic islands while the others are not. Putative horizontal gene transfer events (connected lines) have been compared based on the inconsistency of the amino acid sequence tree (on the left) and the 16S rDNA tree (on the right).

The phylogenies of Phn2 were in accordance with those calculated for the 16S rDNAs (Figure S4). However, Phn1 showed some conflicts (Figure 4). A Phn1 (*A. faecalis* NCIB 8687) from β-*Proteobacteria* clustered with the α-*Proteobacteria*. The *phn*2 loci were usually arranged as *phnCDEE*' and located in the vicinity of other phosphonate utilizing genes, such as *phnFGHIJKLMNOP* (Jochimsen et al., 2011). The *phn*1 locus *A. faecalis* NCIB 8687 was arranged as *phnDCEE*', which had no other functional related genes in vicinity. Thus, the *phn*1 and *phn*2 may be functional different operons.

## Discussion

This study provides a comprehensive analysis of most of the available full-length AioBA sequences. Large scale scanning of the sequences in the vicinity of *aioBA* operons revealed the frequent occurrence of genes related to arsenic and phosphorous metabolism, such as the regulatory *aioXSR* operon and *pst*, *phn*, and *ars* operons (Silver and Phung, 2005). Considering gene synteny and structural analogies between arsenate and phosphate, we presumed that these genes function together in helping these microbes to be able to use even low concentrations of phosphorus needed for vital functions under high concentrations of arsenic, and defined these sequences as the arsenic islands. The *aioBA* operons function to convert AsIII to the less toxic AsV but frequently also use this as a chemolithotrophic energy source. In contrast, *ars* operons are responsible for arsenic efflux after arsenate reduction and have a purely protective role (Silver and Phung, 2005). We found that some strains contain *pst*1 or *phn*1 operons encoding putative phosphate and phosphonate uptake transport systems in the vicinity of the *aio* operons in addition to the distantly located *pst*2 or *phn*2 operons, which raises the question about the functional role of *pst*1 and *phn*1 operons. Previous results indicated that arsenate can increase the *V*_max_ of Pst2 for phosphate uptake (Moreno-Sanchez et al., 2012). AsIII may induce phosphate starvation as a competitive inhibitor of phosphate uptake, and cells may need to express more of these transporters or possibly more specific transporters for phosphate uptake. Similarly, we conjecture that the AsV generated by the AioBA may lead to phosphate starvation and the *pst*1 and *phn*1 may encode additional more specific uptake systems for P assimilation. This proposition is in accordance with the transcriptional profile of *H. arsenicoxydans* ULPAs1, in which *pst*1 operon was induced under conditions of As exposure (Cleiss-Arnold et al., 2010). Recently, it was reported that a *pst*1-like protein discriminated P from AsV 500–850-fold in phosphate-limited condition (Elias et al., 2012). In addition, one could envision PstS1 transporting AsV into the cells and deposited into acidicalcisomes or as part of polyphosphate granules. This had been suggested by Moreno-Sanchez et al. (2012).

In this study, we analyzed the localization of AsIII oxidation genes and found that *aioBA* of the α-*Proteobacteria* was prevalently localized on plasmids. As many arsenic islands were localized on plasmids, we predict that plasmids played a role in the widespread distribution of *aioBA*. Most of the *aioBA* sequences analyzed here could be retrieved from *Proteobacteria* and these sequences could be assigned to two groups, α-*Proteobacteria* and β-/γ-*Proteobacteria*, consistent with a previous analysis (Hamamura et al., 2009). The AioAs generally showed similar phylogeny as their 16S rDNA sequences (Figure 2) indicating an ancient origin of the enzyme (Cai et al., 2009b; Zhou and Xu, 2010). However, several strains showed putative HGT events with AioAs (Figure 2; Table 1) suggesting HGT also play a role during inheritance process (Arsène-Ploetze et al., 2010; Heinrich-Salmeron et al., 2011).

**Table 1:** **Prediction of putative horizontal gene transfer events in the arsenic islands**.

**Strain**	***aioBA***	***aioXSR***	***pst*1**	***phh*1**	***arsB***	***acr3***
**α-PROTEOBACTERIA**						
■ *Agrobacterium tumefaciens* 5A	+	+	+	+		+
■ *Agrobacterium* sp. GW4	+	+	+	+		+
■ *Sinorhizobium* sp. M14	+	+	+	+		+
■ *Nitrobacter hamburgensis* X14	+					
■ *Roseomonas cervicalis* ATCC 49957						
*Acidiphilum multivorum* AIU301 chromosome	+Δ				+	
■ *Acdidiphilium multivorum* AIU301 pACMV2	+Δ				+	
■ *Acdidiphilium* sp. PM	+Δ				+	
**β-PROTEOBACTERIA**						
*Hermniimonas arsenicoxydans* ULPAs1	+	+	+Δ		+	
■ *Achromobacter arsenitoxydans* SY8	+	+	+Δ		+	
■ *Alcaligenes faecalis* NCIB 8687	+	+	+Δ	+Δ	+Δ	

*+ Represents the genes present in the arsenic island; △ Represents putative horizontal gene transfer event suggested by phylogenetic analysis or the presence of transposon elements; ■ Represents the plasmid origin of the genes*.

**Figure 5:**
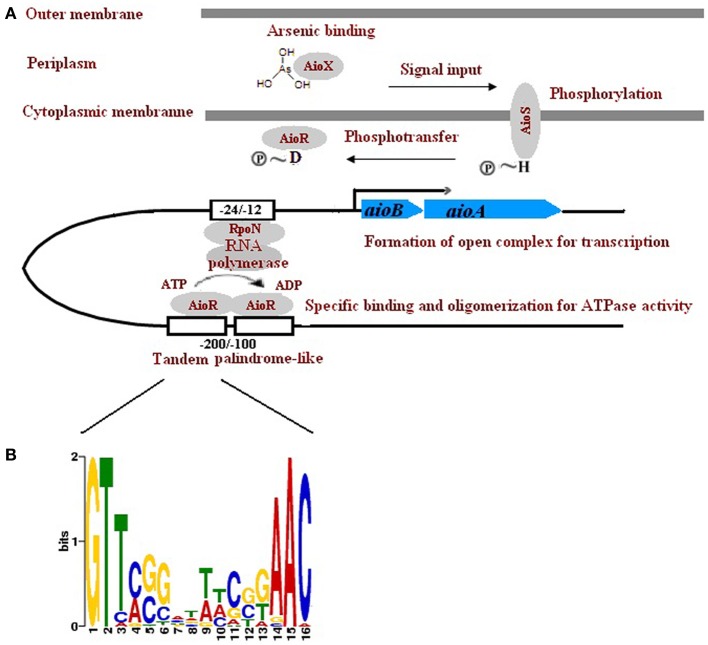
**A proposed mechanism *aioXSR* mediated *aioBA* regulation. (A)** AioX binds to arsenite and delivers the signal to the AioS/AioR two-component system. The phosphorylated AioR binds to the palindrome-like sequence, leading to oligomerization for ATPase activity. Energy conserved by AioR would open the RopN-RNA polymerase complex, and initiate the *aioBA* transcription. **(B)** Sequence logo of palindrome-like sequences located upstream of *aioBA*. Higher bit scores indicate more conservation at respective site.

Unlike *aioBAs*, which are widely distributed among *Proteobacteria*, *Chlorobi*, *Deinococcus-Thermus*, *Chloroflexi*, and even *Archaea*, the three component regulator genes genes *aioXSR* were only found in *Proteobacteria* and displayed opposite transcriptional orientation between α- and β-*Proteobacteria*. It was possible that the *aioXSR* genes emerged in *Proteobacteria* after the introduction of *aioBA*. The regulation of these *aioBA* operons with no *aioXSR* genes is not clear, but they may be controlled by distantly located regulators, or quorum sensing, as proposed by Kashyap et al. (Kashyap et al., 2006). Thus, the regulatory genes *aioXSR* may have evolved independently from *aioBA*. In a few strains including *A*. *tumefaciens* 5A, AioSR regulation of *aioBA* was RpoN-dependent, and the -24/-12 region for RpoN (σ^54^ factor for RNA polymerase) binding was also detected (Kang et al., 2012). The arsenite oxidase regulator AioR belonged to the NtrC family indicating that *aioBA* may be under the regulation of RpoN-dependent σ^54^-type promoter. However, the molecular details of AioR interacting with the promoter, and of the RpoN-RNA polymerase complex initiating transcription are still not known. Here we identified two tandem repeats of palindrome-like sequences which are located 100–200 nt upstream of the *aioB* start codon (Figure 5). The palindrome-like sequences are probably the upstream activating sequences (UAS) of σ^54^-type promoters which function in binding of AioR. The two palindromes and the -24/-12 regions were detected in all of the 12 *aioBA* operons that contained the *aioXSR* three-component system, but absent in other *aioBA* operons without *aioXSR*. Thus, we have to propose that the *aioBAs* without the upstream sequences of *aioXSR* are regulated differently.

The *ars*, *pst*, and *phn* operons were frequently detected on the arsenic gene islands but did not display a similar arrangement in various strains. Some plasticity was found even in the taxonomically closely related strains *A. tumefaciens* 5A, *Agrobacterium* sp. GW4 and *Sinorhizobium* sp. M14. These strains shared the same arrangement in *aio*, *pst*, and *phn* operons, but not in *ars* operons. The large scale synteny of *aio*, *pst* and *phn* operons in these three strains may be due to vertical inheritance, while *ars* operons were integrated independently into the arsenic islands.

The *ars* operons encoded either ArsB or ACR3 as the AsIII efflux pump but did not display the same arrangement of the remaining genes such as *arsC* or *arsH*. This does not indicate a common origin of the different *ars* operons on the respective arsenic islands. The phylogenetic relatedness of ArsB or ACR3 seems to be in accordance with the corresponded tree predicted by 16S rDNA comparison. This suggests that ArsB or ACR3 were both mostly vertically inherited from the gene pool of the respective taxonomic clade. Vertical inheritance and HGT may have contributed to the origin of *pst*1 and *phn*1 operons (Table 1). It is therefore likely the arsenic islands did not evolve as a whole unit but formed independently by acquisition of functionally related genes and operons in respective strains. The elucidation of the phylogeny and distribution of *aio* genes might provide further insight into the evolution of the *aioBA* operon, and lead to better understanding of the arsenic island.

## Methods

### Data sources

The amino acids sequence of AioA from *Agrobacterium tumefaciens* 5A was used as the initial query for a BLASTP search at the National Center for Biotechnology Information (http://www.ncbi.nlm.nih.gov). Partial AioA sequences obtained from degenerate primers were ignored, as there was usually no flanking sequence information for them. We selected the full-length AioA sequences with the following threshold: sequence identity >30%, coverage >80%, starting with methionine and harboring the conserved domain TIGR02693 specific for arsenite oxidase. The selected BLASTP hits were used as query sequences for additional BLASTP searches, until no more full-length AioA hits were found. The corresponding nucleotide sequences where *aioA* was located, as well as the gene annotation information were downloaded in GenBank format for further analysis.

### Detection of gene synteny in the arsenic islands

The GenBank formatted sequences containing 57 *aioA* genes were loaded in the CLC sequence viewer program (http://www.clcbio.com). And the downstream and upstream sequences were scanned over 100 kb. Twenty-one sequences were found in vicinity of *aioBA* which were called arsenic islands, the others are single *aioBA*. The genes in the arsenic islands were exported as image files with the same genes represented by the same colors to detect synteny.

### Phylogenetic analysis of nucleotide or amino acid sequences

All of the gene sequences were searched in the GenBank using the *aioA* sequence and a neighbor-joining (NJ) phylogenetic tree was constructed using ClustalX analysis (Thompson et al., 1997) and MEGA 4.0 software (Tamura et al., 2007). The parameters are as follows: phylogeny test and options (Bootstrap, 1000 replicates), Gaps/Missing Data (Pairwise Deletion), Substitution Model (Poisson correction for amino acids, Kimura 2P for nucleotides). Later on, other phylogenetic comparisons were made using the some methods for 16S rDNA, ArsB, Acr3, pstS, phnC, and the sequences were extracted from the corresponding genomes or other related genomes when necessary.

### Predicting of the chromosome and plasmid location of *aioA* genes

The information on chromosome or plasmid location for all the 21 *arsenic gene islands*, if existing, was identified from the strain notes in GenBank. However, many of the 21 arsenic islands had no information on chromosome or plasmid location because they were from draft genomes. We predicted the chromosome and plasmid location of all the 21 “arsenic islands” by the cBar program (Zhou and Xu, 2010). The cBar program was developed for classifying metagenomes into chromosomal and plasmid sequences based on their different nucleotide pentamer frequencies.

### Detection of conserved sequence motifs

The upstream 300 bp sequences of all the 57 *aioBA* genes were selected. The conserved motifs were detected by The MEME Suite motif-based sequence analysis tools (http://meme.sdsc.edu/meme/intro.html). The sequence logo which graphically represents the sequence conservation was also automatically generated by MEME on-line program.

## Authors' contributions

Hang Li carried out data collection, participated in the bioinformatic analyses and wrote the draft of the manuscript. Mingshun Li participated in bioinformatic analyses and helped to draft the manuscript. Yinyan Huang participated in sequence alignment study. Christopher Rensing participated in the design of the study and drafted the manuscript. Gejiao Wang coordinated the study, participated in its design and wrote the draft of the manuscript. All authors read and approved the final manuscript. We thank Dr. Timothy McDermott for discussion of the study design, editing, and comments on the manuscript.

## Conflict of interest statement

The authors declare that the research was conducted in the absence of any commercial or financial relationships that could be construed as a potential conflict of interest.

## Supplementary material

Supplementary Figure S1 **Phylogenetical trees of ArsB and 16S rDNA sequences.** Bold and ^*^symbol represent proteins from the strains of the arsenic islands while the others are not. Phylogenetic relationship have been compared based on the amino acid sequence tree (on the left) and the 16S rDNA tree (on the right).

Supplementary Figure S2 **Phylogenetical trees of ACR3 and 16S rDNA sequences.** Bold and ^*^symbol represent proteins from the strains of the arsenic islands while the others are not. Phylogenetic relationship have been compared based on the amino acid sequence tree (on the left) and a 16S rDNA tree (on the right).

Supplementary Figure S3 **Phylogenetic trees based on PstS2 and 16S rDNA sequences.** Bold and ^*^symbol represent proteins from the strains of the arsenic islands while the others are not. Phylogenetic relationship have been compared based on the inconsistency of the amino acid sequence tree (on the left) and the 16S rDNA tree (on the right).

Supplementary Figure S4 **Phylogenetic trees of PhnC2 and 16S rDNA sequences.** Bold and ^*^symbol represent proteins from the strains of the arsenic islands while the others are not. Phylogenetic relationship have been compared based on the amino acid sequence tree (on the left) and the16S rDNA tree (on the right).

## References

[B1] Achour A. R. Bauda P. Billard P. (2007). Diversity of arsenite transporter genes from arsenic-resistant soil bacteria. Res. Microbiol. 158, 128–137 10.1016/j.resmic.2006.11.006 17258434

[B2] Arsène-Ploetze F. Koechler S. Marchal M. Coppée J. Y. Chandler M. Bonnefoy V. (2010). Structure, function, and evolution of the *Thiomonas* spp. genome. PLoS Genet. 6:e1000859 10.1371/journal.pgen.1000859 20195515 PMC2829063

[B3] Cai L. Liu G. Rensing C. Wang G. (2009a). Genes involved in arsenic transformation and resistance associated with different levels of arsenic-contaminated soils. BMC Microbial. 9:4 10.1186/1471-2180-9-4 19128515 PMC2631446

[B4] Cai L. Rensing C. Li X. Wang G. (2009b). Novel gene clusters involved in arsenite oxidation and resistance in two arsenite oxidizers: *Achromobacter* sp. SY8 and *Pseudomonas* sp. TS44. Appl. Microbiol. Biotechnol. 83, 715–725 10.1007/s00253-009-1929-4 19283378

[B5] Cleiss-Arnold J. Koechler S. Proux C. Fardeau M. L. Dillies M. A. Coppee J. Y. (2010). Temporal transcriptomic response during arsenic stress in *Herminiimonas arsenicoxydans*. BMC Genomics 11:709 10.1186/1471-2164-11-709 21167028 PMC3022917

[B6] Elias M. Wellner A. Goldin-Azulay K. Chabriere E. Vorholt J. A. Erb T. J. (2012). The molecular basis of phosphate discrimination in arsenate-rich environments. Nature 491, 134–137 10.1038/nature11517 23034649

[B7] Hamamura N. Macur R. E. Korf S. Ackerman G. Taylor W. P. Kozubal M. (2009). Linking microbial oxidation of arsenic with detection and phylogenetic analysis of arsenite oxidase genes in diverse geothermal environments. Environ. Microbiol. 11, 421–431 10.1111/j.1462-2920.2008.01781.x 19196273

[B8] Hao X. Lin Y. Johnstone L. Liu G. Wang G. Wei G. (2012). Genome sequence of the arsenite-oxidizing strain *Agrobacterium tumefaciens* 5A. J. Bcateriol. 194, 903 10.1128/JB.06585-11 22275101 PMC3272953

[B9] Heinrich-Salmeron A. Cordi A. Brochier-Armanet C. Halter D. Pagnout C. Abbaszadeh-fard E. (2011). Unsuspected diversity of arsenite-oxidizing bacteria as revealed by widespread distribution of the *aoxB* gene in prokaryotes. Appl. Environ. Microbiol. 77, 4685–4692 10.1128/AEM.02884-10 21571879 PMC3127681

[B10] Hsieh Y. J. Wanner B. L. (2010). Global regulation by the seven-component Pi signaling system. Curr. Opin. Microbiol. 13, 198–203 10.1016/j.mib.2010.01.014 20171928 PMC2847643

[B11] Huang Y. Li H. Rensing C. Zhao K. Johnstone L. Wang G. (2012). Genome sequence of the facultative anaerobic arsenite-oxidizing and nitrate-reducing bacterium *Acidovorax* sp. strain NO1. J. Bacteriaol. 194, 1635–1636 10.1128/JB.06814-11 22374962 PMC3294833

[B12] Inskeep W. P. Macur R. E. Hamamura N. Warelow T. P. Ward S. A. Santini J. M. (2007). Detection, diversity and expression of aerobic bacterial arsenite oxidase genes. Environ. Microbiol. 9, 934–943 10.1111/j.1462-2920.2006.01215.x 17359265

[B13] Jochimsen B. Lolle S. McSorley F. R. Nabi M. Stougaard J. Zechel D. L. (2011). Five phosphonate operon gene products as components of a multi-subunit complex of the carbon-phosphorus lyase pathway. Proc. Natl. Acad. Sci. U.S.A.. 108, 11393–11398 10.1073/pnas.1104922108 21705661 PMC3136323

[B14] Kang Y. S. Bothner B. Rensing C. McDermott T. R. (2012). Involvement of RpoN in regulating bacterial arsenite oxidation. Appl. Environ. Microbiol. 78, 5638–5645 10.1128/AEM.00238-12 22660703 PMC3406167

[B15] Kashyap D. R. Botero L. M. Franck W. L. Hassett D. J. McDermott T. R. (2006). Complex regulation of arsenite oxidation in *Agrobacterium tumefaciens*. J. Bacteriol. 188, 1081–1088 10.1128/JB.188.3.1081-1088.2006 16428412 PMC1347330

[B16] Koechler S. Cleiss-Arnold J. Proux C. Sismeiro O. Dillies M. A. Goulhen-Chollet F. (2010). Multiple controls affect arsenite oxidase gene expression in *Herminiimonas arsenicoxydans*. BMC Microbiol. 10:53–65 10.1186/1471-2180-10-53 20167112 PMC2848651

[B17] Li X. Hu Y. Gong J. Lin Y. Johnstone L. Rensing C. (2012). Genome sequence of the highly efficient arsenite-oxidizing bacterium *Achromobacter arsenitoxydans* SY8. J. Bacteriol. 194, 1243–1244 10.1128/JB.06667-11 22328747 PMC3294760

[B18] Lieutaud A. van Lis R. Duval S. Capowiezl L. Muller D. Lebrun R. (2010). Arsenite oxidase from *Ralstonia* sp. 22: characterization of the enzyme and its interaction with soluble cytochromes. J. Biol. Chem. 285, 20433–20441 10.1074/jbc.M110.113761 20421652 PMC2898339

[B19] Lin Y. Fan H. Hao X. Johnstone L. Hu Y. Wei G. (2012). Draft genome sequence of *Halomonas* sp. strain HAL1, a moderately halophilic arsenite-oxidizing bacterium isolated from gold-mine soil. J. Bacteriol. 194, 199–200 10.1128/JB.06359-11 22156396 PMC3256601

[B20] Liu G. Liu M. Kim E. H. Matty W. Bothner B. Lei B. (2012). A periplasmic arsenite-binding protein involved in regulating arsenite oxidation. Environ. Microbiol. 14, 1624–1634 10.1111/j.1462-2920.2011.02672.x 22176720

[B21] Moreno-Sanchez D. Hernandez-Ruiz L. Ruiz F. A. Docampo R. (2012). Polyphosphate is a novel pro-inflammatory regulator of mast cells and is located in acidocalcisomes. J. Biol. Chem. 287, 28435–28444 10.1074/jbc.M112.385823 22761438 PMC3436523

[B22] Muller D. Médigue C. Koechler S. Barbe V. Barakat M. Talla E. (2007). A tale of two oxidation states: bacterial colonization of arsenic-rich environments. PLoS Genet. 3:e53 10.1371/journal.pgen.0030053 17432936 PMC1851979

[B23] Phung L. T. Trimble W. L. Meyer F. Gilbert J. A. Silver S. (2012). Draft genome sequence of *Alcaligenes faecalis* subsp. faecalis NCIB 8687 (CCUG 2071). J. Bacteriol. 194, 5153 10.1128/JB.01185-12 22933773 PMC3430353

[B24] Quéméneur M. Heinrich-Salmeron A. Muller D. Lièvremont D. Jauzein M. Bertin P. N. (2008). Diversity surveys and evolutionary relationships of *aoxB* genes in aerobic arsenite-oxidizing bacteria. Appl. Environ. Microbiol. 74, 4567–4573 10.1128/AEM.02851-07 18502920 PMC2493162

[B25] Richey C. Chovanec P. Hoeft S. E. Oremland R. S. Basu P. Stolz J. F. (2009). Respiratory arsenate reductase as a bidirectional enzyme. Biochem. Biophys. Res. Commun. 382, 298–302 10.1016/j.bbrc.2009.03.045 19285953

[B26] San Martin-Uriz P. Gomez M. J. Arcas A. Bargiela R. Amils R. (2011). Draft genome sequence of the Electricigen *Acidiphilium* sp. strain PM (DSM 24941). J. Bacteriol. 193, 5585–5586 10.1128/JB.05386-11 21914891 PMC3187463

[B27] Sardiwal S. Santini J. M. Osborne T. H. Djordjevic S. (2010). Characterization of a two-component signal transduction system that controls arsenite oxidation in the chemolithoautotroph NT-26. FEMS Microbiol. Lett. 313, 20–28 10.1111/j.1574-6968.2010.02121.x 21039781

[B28] Shingler V. (2011). Signal sensory systems that impact σ^54^-dependent transcription. FEMS Microbiol. Rev. 35, 425–440 10.1111/j.1574-6976.2010.00255.x 21054445

[B29] Silver S. Phung L. T. (2005). Genes and enzymes involved in bacterial oxidation and reduction of inorganic arsenic. Appl. Environ. Microbiol. 71, 599–608 10.1128/AEM.71.2.599-608.2005 15691908 PMC546828

[B30] Tamura K. Dudley J. Nei M. Kumar S. (2007). MEGA4: Molecular Evolutionary Genetics Analysis (MEGA) software version 4.0. Mol. Biol. Evol. 24, 1596–1599 10.1093/molbev/msm092 17488738

[B31] Thompson J. D. Gibson T. J. Plewniak F. Jeanmougin F. Higgins D. G. (1997). The CLUSTAL_X windows interface: flexible strategies for multiple sequence alignment aided by quality analysis tools. Nucleic Acids Res. 25, 4876–4882 10.1093/nar/25.24.4876 9396791 PMC147148

[B32] Trimble W. L. Phung L. T. Meyer F. Gilbert J. A. Silver S. (2012a). Draft genome sequence of *Agrobacterium albertimagni* strain AOL15. J. Bacteriol. 194, 6986–6987 10.1128/JB.01912-12 23209236 PMC3510623

[B33] Trimble W. L. Phung L. Meyer F. Silver S. Gilbert J. A. (2012b). Draft genome sequence of *Achromobacter piechaudii* strain HLE. J. Bacteriol. 194, 6355 10.1128/JB.01660-12 23105084 PMC3486377

[B34] Tuffin I. M. de Groot P. Deane S. M. Rawlings D. E. (2005). An unusual Tn21-like transposon containing an *ars* operon is present in highly arsenic-resistant strains of the biomining bacterium *Acidithiobacillus caldus*. Microbiology 151, 3027–3039 10.1099/mic.0.28131-0 16151213

[B35] Zargar K. Conrad A. Bernick D. L. Lowe T. M. Stolc V. Hoeft S. (2012). ArxA, a new clade of arsenite oxidase within the DMSO reductase family of molybdenum oxidoreductases. Environ. Microbiol. 14, 1635–1645 10.1111/j.1462-2920.2012.02722.x 22404962

[B36] Zargar K. Hoeft S. Oremland R. Saltikov C. W. (2010). Identification of a novel arsenite oxidase gene, *arxA*, in the haloalkaliphilic, arsenite-oxidizing bacterium *Alkalilimnicola ehrlichii* strain MLHE-1. J. Bacteriol. 192, 3755–3762 10.1128/JB.00244-10 20453090 PMC2897359

[B37] Zhou F. Xu Y. (2010). cBar: a computer program to distinguish plasmid-derived from chromosome-derived sequence fragments in metagenomics data. Bioinformatics 26, 2051–2052 10.1093/bioinformatics/btq299 20538725 PMC2916713

