# Supplementary figures and images for: In silico analysis of bacterial arsenic islands reveals remarkable synteny and functional relatedness between arsenate and phosphate
Source: Front Microbiol. 2013 Nov 20;4:347. doi: 10.3389/fmicb.2013.00347 (PMC3834237; doi:10.3389/fmicb.2013.00347)

\*

# ArsB

# 16S rDNA

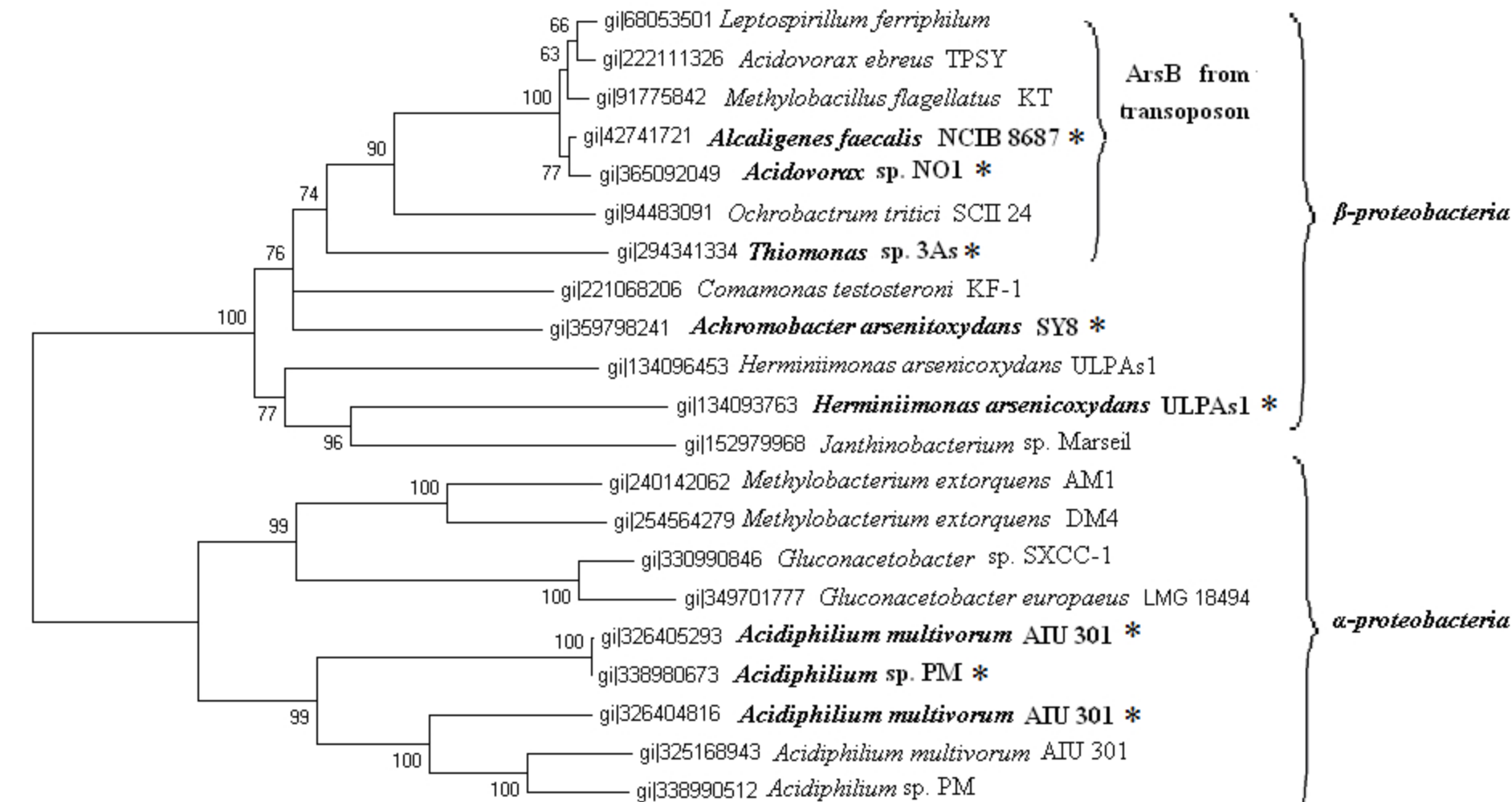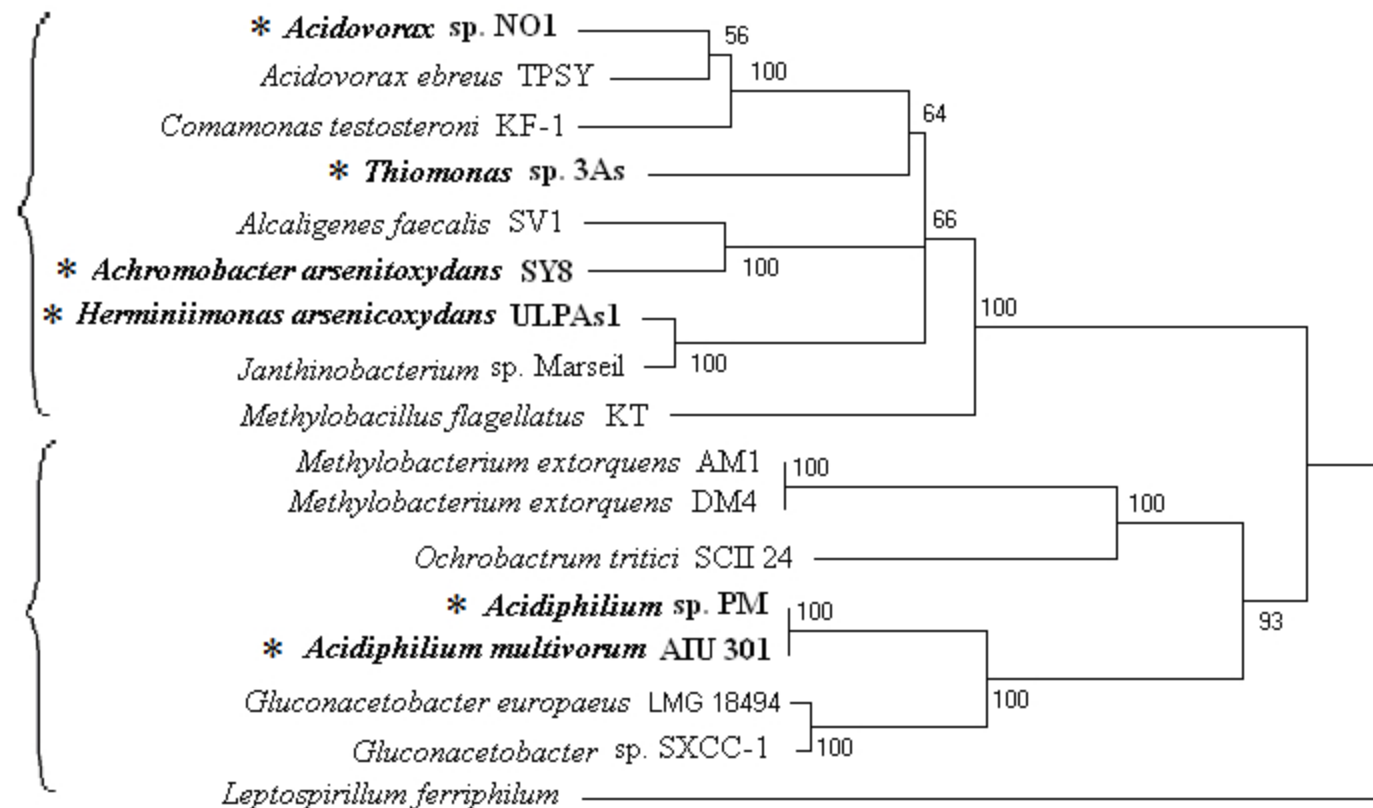

Supplement: Supplementary Figure S1 — Phylogenetical trees of ArsB and 16S rDNA sequences. Bold and *symbol represent proteins from the strains of the arsenic islands while the others are not. Phylogenetic relationship have been compared based on the amino acid sequence tree (on the left) and the 16S rDNA tree (on the right). [file Presentation1.PDF]

# PstS2

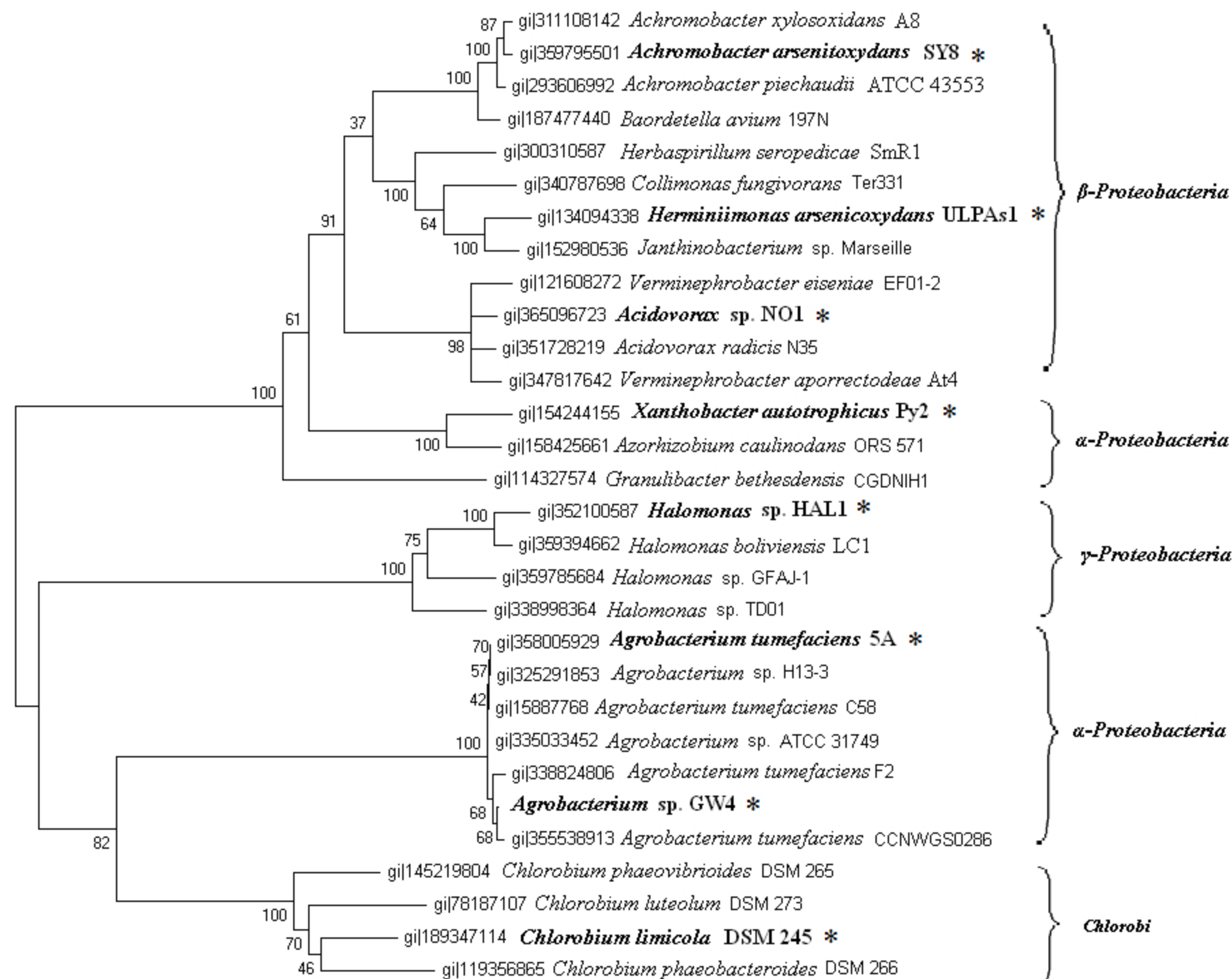

0.2

# 16S rDNA

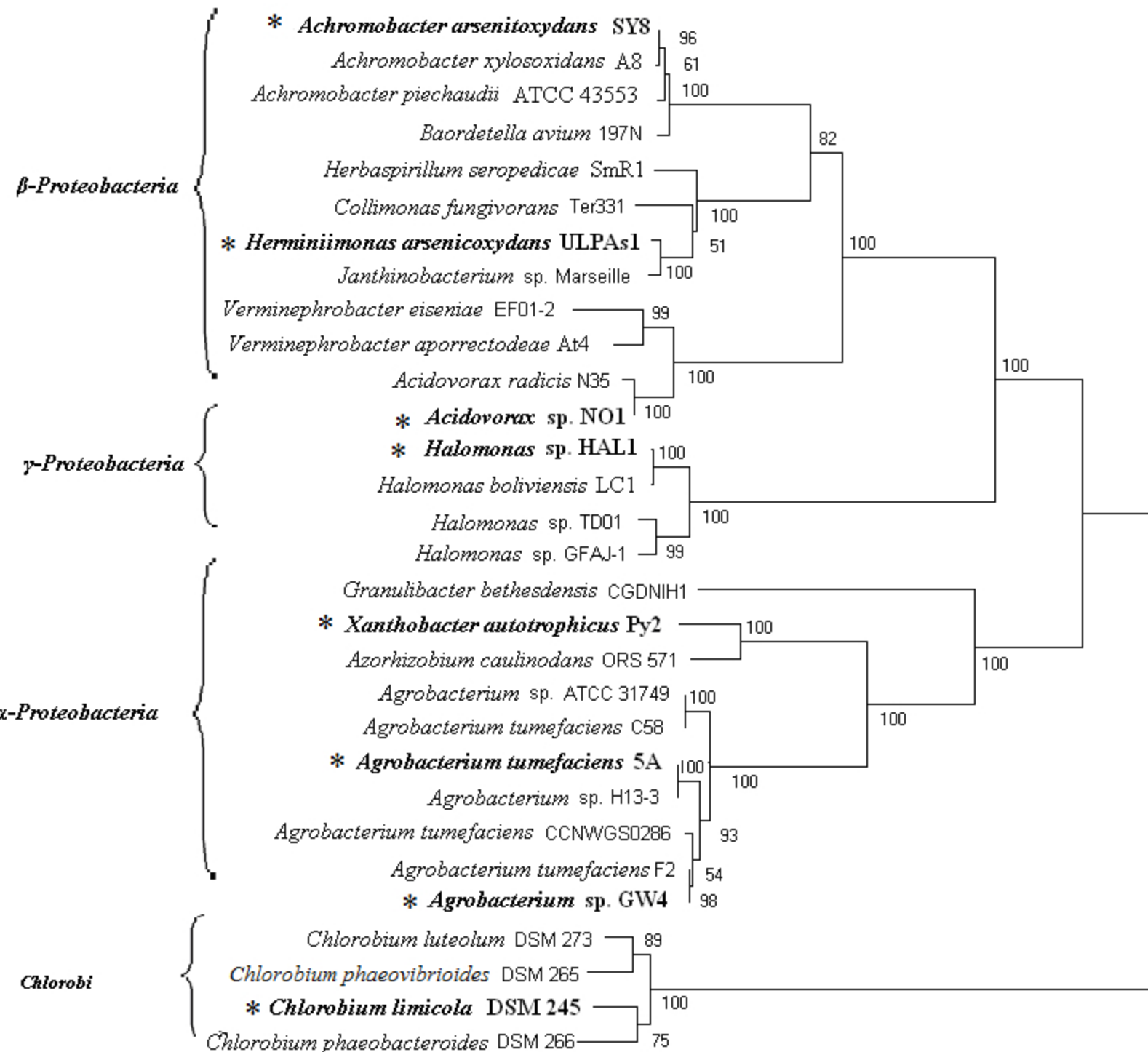

0.02

Supplement: Supplementary Figure S3 — Phylogenetic trees based on PstS2 and 16S rDNA sequences. Bold and *symbol represent proteins from the strains of the arsenic islands while the others are not. Phylogenetic relationship have been compared based on the inconsistency of the amino acid sequence tree (on the left) and the 16S rDNA tree (on the right). [file Presentation3.PDF]

## PhnC2

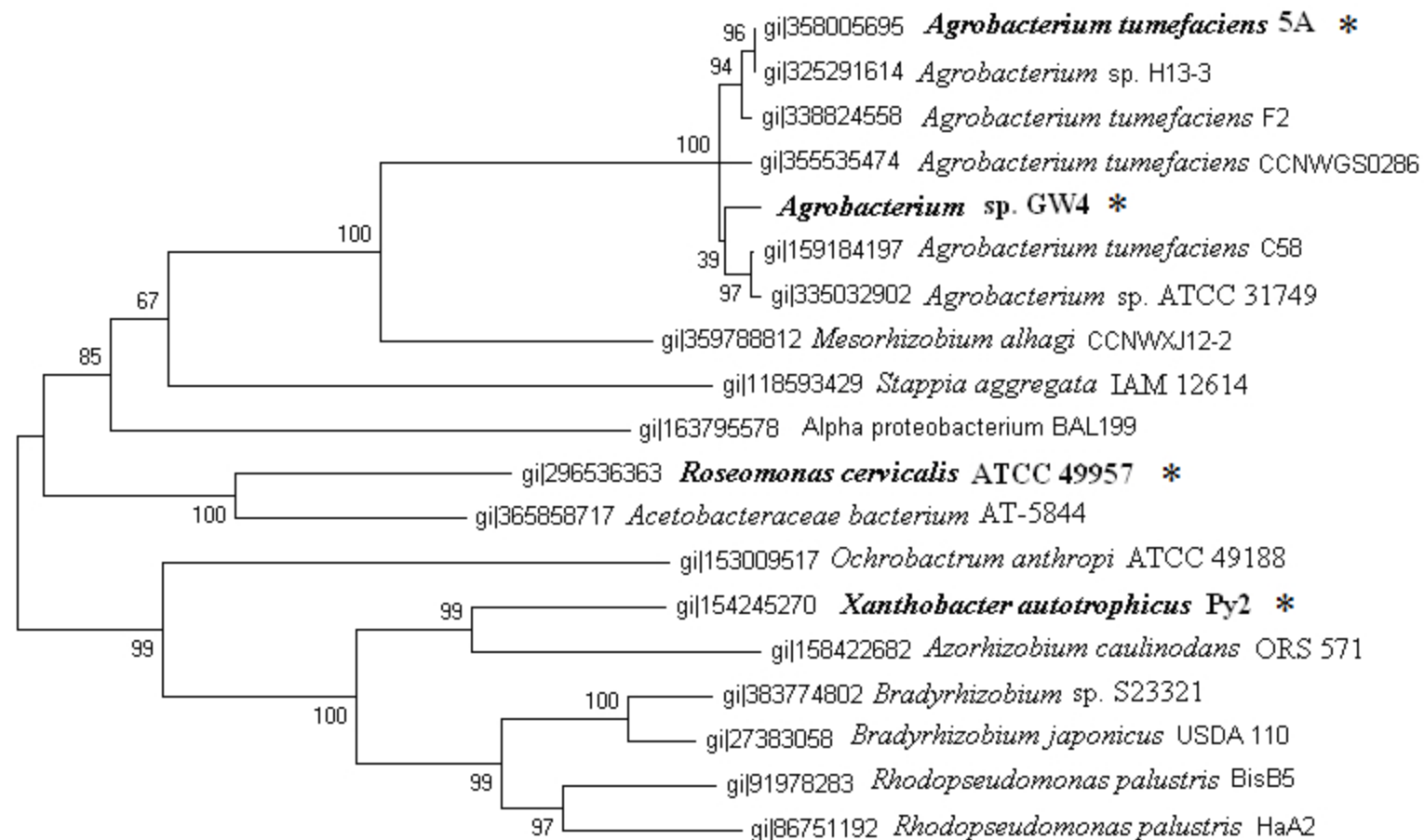

## 16S rDNA

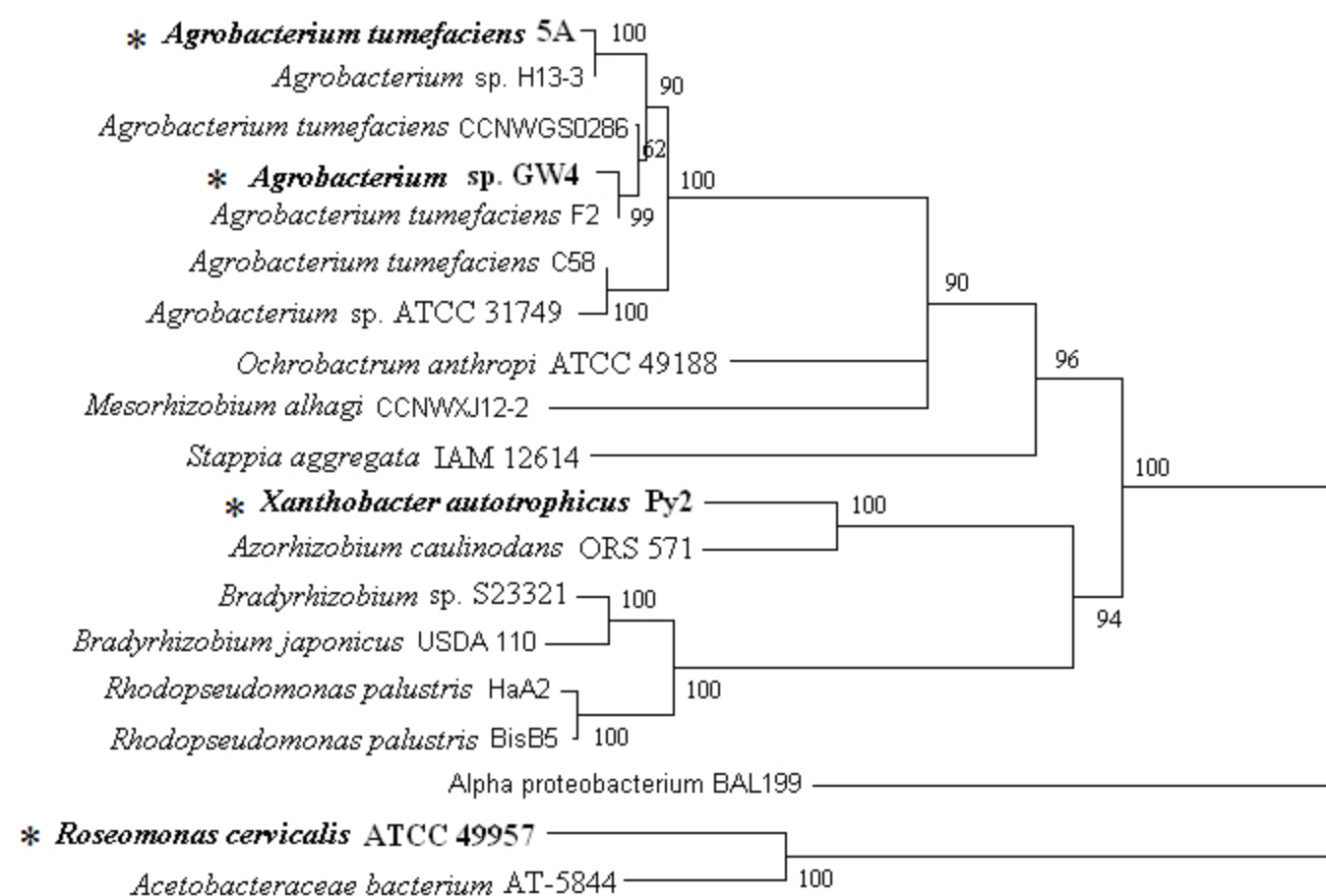

Supplement: Supplementary Figure S4 — Phylogenetic trees of PhnC2 and 16S rDNA sequences. Bold and *symbol represent proteins from the strains of the arsenic islands while the others are not. Phylogenetic relationship have been compared based on the amino acid sequence tree (on the left) and the16S rDNA tree (on the right). [file Presentation4.PDF]
