# Supplementary material for: In silico analysis of bacterial arsenic islands reveals remarkable synteny and functional relatedness between arsenate and phosphate
Source: Front Microbiol. 2013 Nov 20;4:347. doi: 10.3389/fmicb.2013.00347 (PMC3834237; doi:10.3389/fmicb.2013.00347)
Supplement: Supplementary Figure S2 — Phylogenetical trees of ACR3 and 16S rDNA sequences. Bold and *symbol represent proteins from the strains of the arsenic islands while the others are not. Phylogenetic relationship have been compared based on the amino acid sequence tree (on the left) and a 16S rDNA tree (on the right). [file Presentation2.PDF]

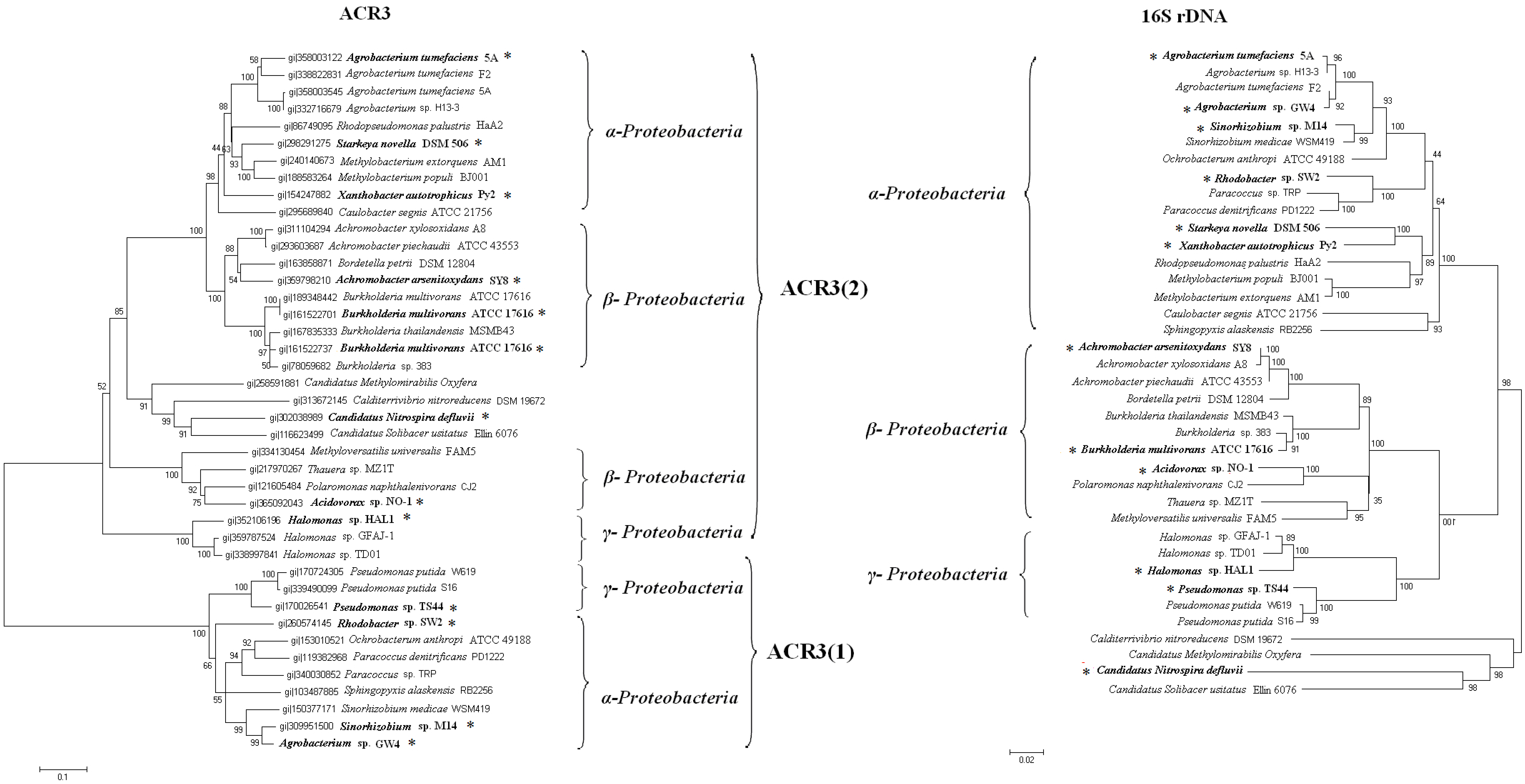
ACR3
gi|358003122 Agrobacterium tumefaciens 5A *
gi|338822831 Agrobacterium tumefaciens F2
gi|358003545 Agrobacterium tumefaciens 5A
gi|332716679 Agrobacterium sp. H13-3
gi|86749095 Rhodopseudomonas palustris HaA2
gi|298291275 Starkeya novella DSM 506 *
gi|240140673 Methylobacterium extorquens AM1
gi|188583264 Methylobacterium populi BJ001
gi|154247882 Xanthobacter autotrophicus Py2 *
gi|295689840 Caulobacter segnis ATCC 21756
gi|311104294 Achromobacter xylosoxidans A8
gi|293603687 Achromobacter piechaudii ATCC 43553
gi|163858871 Bordetella petrii DSM 12804
gi|359798210 Achromobacter arsenitoxydans SY8 *
gi|189348442 Burkholderia multivorans ATCC 17616
gi|161522701 Burkholderia multivorans ATCC 17616 *
gi|167835333 Burkholderia thailandensis MSMB43
gi|161522737 Burkholderia multivorans ATCC 17616 *
gi|78059682 Burkholderia sp. 383
gi|258591881 Candidatus Methylomirabilis Oxyfera
gi|313672145 Calditerrivibrio nitroreducens DSM 19672
gi|302038989 Candidatus Nitrospira defluvii *
gi|116623499 Candidatus Solibacter usitatus Ellin 6076
gi|334130454 Methyloversatilis universalis FAM5
gi|217970267 Thauera sp. MZ1T
gi|121605484 Polaromonas naphthalenivorans CJ2
gi|365092043 Acidovorax sp. NO-1 *
gi|352106196 Halomonas sp. HAL1 *
gi|359787524 Halomonas sp. GFAJ-1
gi|338997841 Halomonas sp. TD01
gi|170724305 Pseudomonas putida W619
gi|339490099 Pseudomonas putida S16
gi|170026541 Pseudomonas sp. TS44 *
gi|260574145 Rhodobacter sp. SW2 *
gi|153010521 Ochrobacterum anthropi ATCC 49188
gi|119382968 Paracoccus denitrificans PD1222
gi|340030852 Paracoccus sp. TRP
gi|103487885 Sphingopyxis alaskensis RB2256
gi|150377171 Sinorhizobium medicae WSM419
gi|309951500 Sinorhizobium sp. M14 *
Agrobacterium sp. GW4 *
α-Proteobacteria
β- Proteobacteria
β- Proteobacteria
γ- Proteobacteria
γ- Proteobacteria
α-Proteobacteria
ACR3(2)
ACR3(1)
0.1
16S rDNA
* Agrobacterium tumefaciens 5A
Agrobacterium sp. H13-3
Agrobacterium tumefaciens F2
* Agrobacterium sp. GW4
* Sinorhizobium sp. M14
Sinorhizobium medicae WSM419
Ochrobacterum anthropi ATCC 49188
* Rhodobacter sp. SW2
Paracoccus sp. TRP
Paracoccus denitrificans PD1222
* Starkeya novella DSM 506
* Xanthobacter autotrophicus Py2
Rhodopseudomonas palustris HaA2
Methylobacterium populi BJ001
Methylobacterium extorquens AM1
Caulobacter segnis ATCC 21756
Sphingopyxis alaskensis RB2256
* Achromobacter arsenitoxydans SY8
Achromobacter xylosoxidans A8
Achromobacter piechaudii ATCC 43553
Bordetella petrii DSM 12804
Burkholderia thailandensis MSMB43
Burkholderia sp. 383
* Burkholderia multivorans ATCC 17616
* Acidovorax sp. NO-1
Polaromonas naphthalenivorans CJ2
Thauera sp. MZ1T
Methyloversatilis universalis FAM5
Halomonas sp. GFAJ-1
Halomonas sp. TD01
* Halomonas sp. HAL1
* Pseudomonas sp. TS44
Pseudomonas putida W619
Pseudomonas putida S16
Calditerrivibrio nitroreducens DSM 19672
Candidatus Methylomirabilis Oxyfera
* Candidatus Nitrospira defluvii
Candidatus Solibacter usitatus Ellin 6076
α-Proteobacteria
β- Proteobacteria
γ- Proteobacteria
0.02
